# ECLIM-SEHOP: how to develop a platform to conduct academic trials for childhood cancer

**DOI:** 10.1007/s12094-024-03445-0

**Published:** 2024-04-10

**Authors:** Antonio Juan-Ribelles, Francisco Bautista, Adela Cañete, Alba Rubio-San-Simón, Anna Alonso-Saladrigues, Raquel Hladun, Susana Rives, Jose Luís Dapena, Jose María Fernández, Álvaro Lassaletta, Ofelia Cruz, Gemma Ramírez-Villar, Jose Luís Fuster, Cristina Diaz de Heredia, Miguel García-Ariza, Eduardo Quiroga, María del Mar Andrés, Jaime Verdú-Amorós, Antonio Molinés, Blanca Herrero, Mónica López, Catalina Márquez, María Toboso, Frencisco Lendínez, Jose Gómez Sirvent, María Tallón, Guiomar Rodríguez, Tomás Acha, Lucas Moreno, Ana Fernández-Teijeiro, the Sociedad Española de Hematología y Oncología Pediátrica (SEHOP)

**Affiliations:** 1 Pediatric Oncology and Hematology Unit, Hospital Universitari y Politécnic La Fe, Instituto de Investigación Sanitaria La Fe (IISLAFE), Valencia, Spain; 2 Princess Máxima Center for Pediatric Oncology, Utrecht, The Netherlands; 3 Pediatric Oncology, Hematology and Stem Cell Transplant Department, Hospital Infantil Universitario Niño Jesús, Madrid, Spain; 4 Pediatric Cancer Center Barcelona (PCCB), Hospital Sant Joan de Déu, Institut de Recerca San Joan de Déu (IRSJD), Barcelona, Spain; 5 Division of Pediatric Hematology and Oncology, Vall d’Hebron Comprehensive Cancer Center, Hospital Universitari Vall d’Hebrón, Passeig Vall d’Hebron 119, 08172 Barcelona, Spain; 6 Department of Pediatric Oncology, Hospital Universitario Virgen del Rocío, Seville, Spain; 7 Hospital Universitario Virgen de la Arrixaca, Instituto Murciano de Investigación Biosanitaria (IMIB), Murcia, Spain; 8 Biocruces Bizkaia Health Research Institute, Hospital Universitario Cruces, Osakidetza, Barakaldo, Bizkaia Spain; 9 INCLIVA - Biomedical Research Institute, Hospital Clínico Universitario, Valencia, Spain; 10 Complejo Hospitalario Universitario Insular Materno-Infantil, Las Palmas de Gran Canaria, Las Palmas Spain; 11 Hematology Department, University Hospital Marqués de Valdecilla (IDIVAL), Santander, Spain; 12 Unidad de Oncohematologia Pediatrica. Hospital Infantil Universitario Torrecárdenas, Almeria, Spain; 13 Hospital Universitario Nuestra Señora de la Candelaria, Santa Cruz de Tenerife, Spain; 14 Hospital Alvaro Cunqueiro, Vigo, Spain; 15 Sofpromed, CRO para ensayos clínicos en España, Palma de Mallorca, Spain; 16 Unidad de Oncología Pediátrica, Hospital Materno-Infantil Carlos Haya, Málaga, Spain; 17 Hospital Universitario Virgen Macarena, Seville, Spain

**Keywords:** Clinical trials, Pediatric hematology and oncology, Drug development, Clinical research, Metrics

## Abstract

**Introduction:**

ECLIM-SEHOP platform was created in 2017. Its main objective is to establish the infrastructure to allow Spanish participation into international academic collaborative clinical trials, observational studies, and registries in pediatric oncology. The aim of this manuscript is to describe the activity conducted by ECLIM-SEHOP since its creation.

**Methods:**

The platform’s database was queried to provide an overview of the studies integrally and partially supported by the organization. Data on trial recruitment and set-up/conduct metrics since its creation until November 2023 were extracted.

**Results:**

ECLIM-SEHOP has supported 47 studies: 29 clinical trials and 18 observational studies/registries that have recruited a total of 5250 patients. Integral support has been given to 25 studies: 16 trials recruiting 584 patients and nine observational studies/registries recruiting 278 patients. The trials include front-line studies for leukemia, lymphoma, brain and solid extracranial tumors, and other key transversal topics such as off-label use of targeted therapies and survivorship. The mean time from regulatory authority submission to first patient recruited was 12.2 months and from first international site open to first Spanish site open was 31.3 months.

**Discussion:**

ECLIM-SEHOP platform has remarkably improved the availability and accessibility of international academic clinical trials and has facilitated the centralization of resources in childhood cancer treatment. Despite the progressive improvement on clinical trial set-up metrics, timings should still be improved. The program has contributed to leveling survival rates in Spain with those of other European countries that presented major differences in the past.

## Introduction

### Pediatric cancer in Spain

Childhood cancers are considered rare diseases according to the RARECARE European project (incidence < 6 cases per 100,000) [1], but still represents the leading cause of disease-related death in children. In Spain, with a population of 6.6 million children under the age of 14, approximately 1100 new cases of childhood cancer are diagnosed yearly with an incidence rate of 165 cases per million according to the national registry [2]. Further 141 patients per year of cancer in adolescents (15–19 years of age) are also registered [2].

Nonetheless, survival has considerably improved within the last decades and current overall survival in Spain is 82% at 5 years [3, 4]. This figure has increased by 2% in the last 4 years and is gradually equalizing with the survival of other European countries (Germany 82.6%, Austria 85.3%, Belgium 83.8%) (EUROCARE-6 data) [5]. Despite this improvement, there has been limited progress in patient survival for difficult-to-treat pediatric malignancies such as metastatic sarcomas or certain brain tumors such as diffuse midline or pontine gliomas. In addition, childhood cancer survivors still face an unacceptable burden of long-term toxicities and sequelae to conventional therapies that require improvements in risk-adapted strategies and the incorporation of new agents [6].

The aim of SEHOP (Spanish Society of Pediatric Hematology and Oncology) (http://www.sehop.org) [7] is to ensure that every child, adolescent, and young adult is assisted in institutions that have the necessary means and sufficiently qualified health professionals using the same therapeutic schemes that are internationally recognized. In 1979, the Registro Español de Tumores Infantiles (RETI-SEHOP tumor registry) was created as a tool to improve knowledge in incidence and survival in Spain and to date more than 35,000 children with cancer have been registered. According to this registry, there are 46 centers treating childhood cancer in Spain, and only 12 of them treat more than 30 patients per year, the standard defined by SIOPE (European Society for Paediatric Oncology). The geographical dispersion, together with much needed improvement in survival makes the realization of academic clinical trials challenging, as they bring new standards and improve the quality of care through central review of imaging and pathology, and improve treatment compliance through enhanced trial monitoring activities.

## Current status of international pediatric clinical trials in Spain

Notable, improvement has been made in the last years 7 years favoring the access to innovative agents for children and adolescents with cancer and the implementation of academic trials. In 2007, European legislation favored the development of new anti-cancer drugs in children through the pediatric investigation plan (PIP) requirement for pharmaceutical companies [8]. In addition, in 2015, the clinical trial regulations were updated (Royal Decree 1090/2015 of 4 December) [9], which eases the administrative burden and reduces costs for opening new academic studies. These two changes have favored greater access to new cancer therapies in childhood.

Other factors that have contributed to the generalization of academic trials are the active engagement of SEHOP with the adult cancer communities such as the Spanish sarcoma investigation group (GEIS) [10], the active participation within SIOP-Europe working groups and the incorporation of Spanish centers in the ITCC consortium (Innovative Therapies for Children with Cancer) [11, 12]. Finally, the key factor that has promoted the development of academic clinical trials is the implementation of a national pediatric program to facilitate the incorporation of clinical trials in the country: ECLIM (Multicenter international clinical trials).

## Role of the ECLIM-SEHOP platform

ECLIM-SEHOP program was created in 2017 and fully established in 2018 [13, 14] by members of SEHOP. The objectives of the platform are:

1. To establish the infrastructure to allow the participation of Spain into international academic collaborative clinical trials.
2. To increase the number of academic international clinical trials open in the country.
3. To improve the set-up (timelines) and conduct quality control of academic and international clinical trials open in Spain.
4. To undertake trial-related activities as required from the international sponsors and national investigators: Regulatory submissions to competent authority and ethics committees, site contracting, monitoring, pharmacovigilance, management of biological samples, and central pathology and radiology review.

**Fig. 1:**
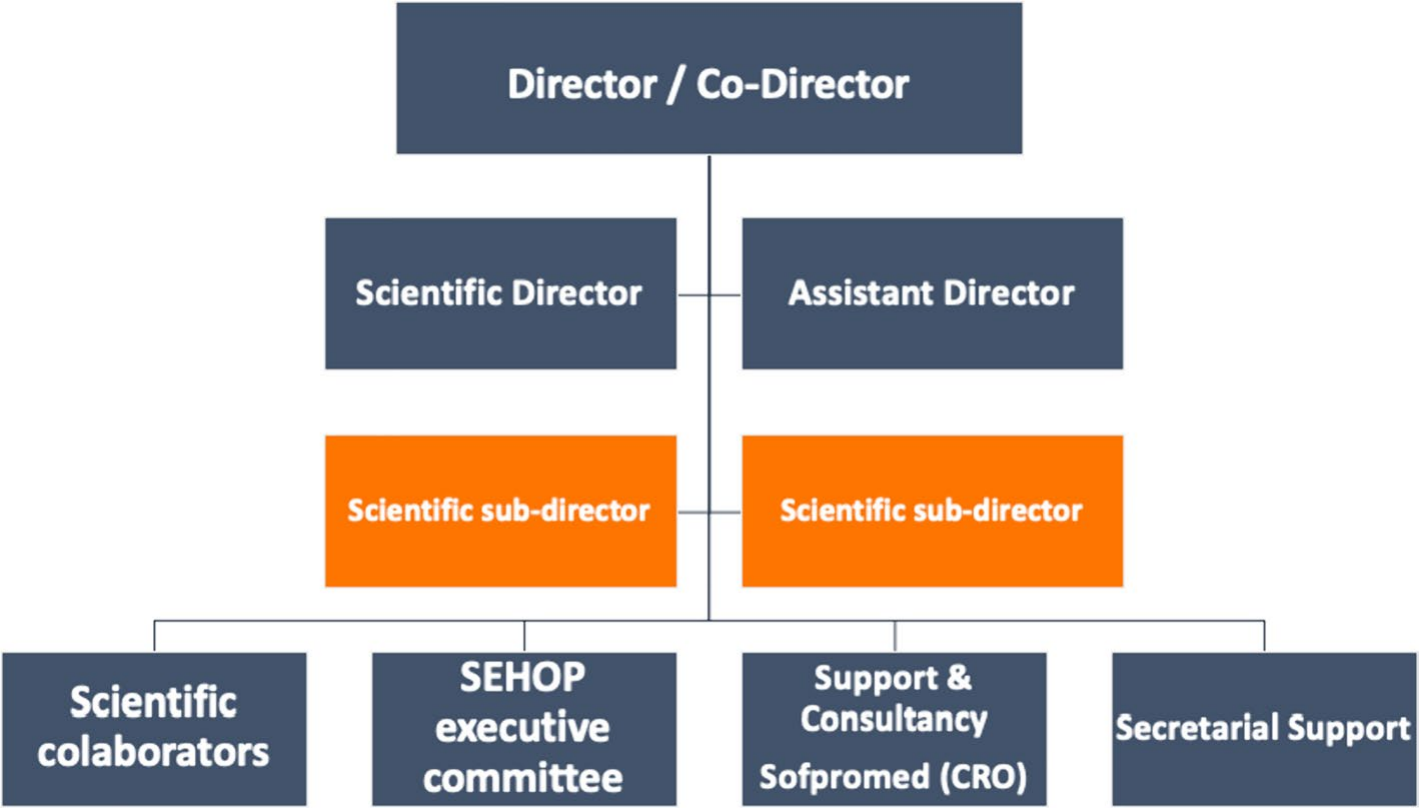
ECLIM-SEHOP structured organigram 2023

To have the capacity and expertise to perform all trial-related tasks required by international sponsors, an agreement with a clinical research organization (CRO) with a solid experience in academic clinical trials was sought and sustained over the years (http://www.sofpromed.com) [15]. Figure 1 shows the hierarchically structured organization of ECLIM-SEHOP supported by four main pillars.

The purpose of this manuscript is to describe the activity conducted by ECLIM-SEHOP focusing on data related to the studies portfolio, infrastructure evolution, recruitment and trial metrics.

## Materials and methods

We provide information on clinical trials, observational studies and registries fully or partially supported by ECLIM-SEHOP from its creation in the beginning of 2017 to the 30 November 2023 including recruitment data. Integral support was defined as all trial-related activities being conducted by the platform. Descriptive metrics of all the activities conducted by the platform including timings regarding the opening, regulatory processes, and set-up procedures of the protocols were revised until 31 May 2023. The organization’s database with support from the CRO was required for this purpose. Descriptive statistics were used.

The timings measured covered the submission to the ethics committee (CEIm), the submission to the Spanish competent authorities (AEMPS), the time from the opening of the first site to the first patient recruited, and the timing from the first international site open to the first Spanish site open.

## Results

### Clinical studies portfolio

ECLIM-SEHOP has provided support to 47 studies from January 2017 to November 2023: 29 clinical trials and 18 observational studies or registries. Integral support has been given to 25 studies (16 clinical trials and 9 observational studies/registries). Partial support has been given to the other 22 studies (13 clinical trials and 9 observational studies/registries).

A relevant contribution has been the creation of the SEHOP-CLOUD imaging platform. This system allows anonymized DICOM medical image uploading and downloading. The web program contributes to the collection of medical images and facilitates central review of imaging and remote consultations. This infrastructure has been regularly used to facilitate centralized revision by expert radiologists in a particular area. It has also been useful to share images between centers to discuss in pediatric tumor boards.

**Fig. 2:**
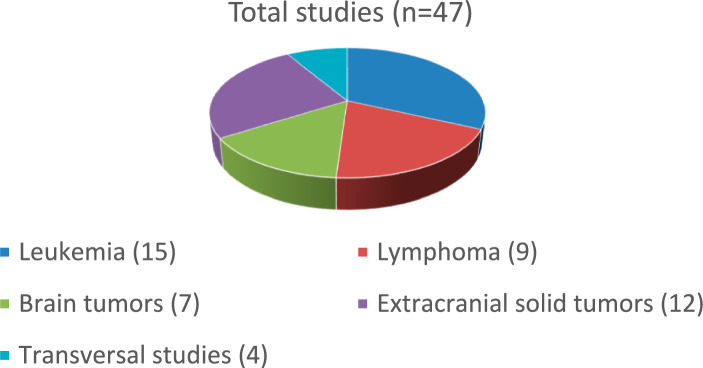
Distribution of ECLIM-SEHOP studies according to cancer type

Table 1 shows all the studies that receive integral support from ECLIM-SEHOP and Table 2 shows the studies partially supported. Figure 2 shows the distribution of the number of studies by type of malignancy.

**Table 1:** Clinical trials, observational studies, and registries integrally supported by ECLIM-SEHOP

Integral support (*n* = 25)		
Clinical trials (*n*=16)	Indication	EudraCT
EURONET PHL-C2	Hodgkin lymphoma	2012-004053-88
NOPHO-DBH-AML 2012	Acute myeloid leukemia	2012-002934-35
IntReALL HR 2010	Relapsed acute lymphoblastic leukemia	2012-000810-12
ALL SCTped Forum 2012	Stem cell transplantation for acute lymphoblastic leukemia	2012-003032-22
SIOP Ependymoma II	Ependymoma	2013-002766-39
LBL-2018	Lymphoblastic lymphoma	2017-001691-39
ESPHALL 2017	Phi + acute lymphoblastic leukemia	2017-000705-20
RANDOMET	Metastasic Wilms tumor	2018-000533-13
FAR-RMS	Rhabdomyosarcoma	2018-000515-24
ALL Together	Acute lymphoblastic leukemia	2018-001795-38
SIOP-ATRT-01	Atypical teratoid rhabdoid tumor	2018-003335-29
SIOP-HR-MB	High-risk medulloblastoma	2018-004250-17
Pro-Teico	Supportive care acute myeloid leukemia	2020-000508-13
INTERFANT-21	Infant acute lymphoblastic leukemia	2021-000213-16
Glo-BNHL	Relapsed B non-Hodgkin lymphoma	2021-004283-10
ALCL—VBL	Anaplastic large cell lymphoma ALK +	2017-002935-40

Observational studies/registries (*n* = 9)	Indication	EudraCT
I-CML-PED Registry	Chronic myeloid leukemia	–
INTERFANT-06	Infant acute lymphoblastic leukemia	–
IBFM AMBI 2018	Ambigous and mixed lineage acute lymphoblastic leukemia	–
UMBRELLA	Wilms tumor and other renal tumors	–
LOGGIC Core	Low grade glioma	–
Survivorship Passport	Pediatric cancer survivors	–
SACHA	All malignancies—compassionate use	–
BIOPORTAL registry	Neuroblastic tumors	–
SEHOP-CLOUD	All solid tumors—central review imaging platform	–

**Table 2:** Clinical trials, observational studies, and registries partially supported by ECLIM-SEHOP

Partial support (*n* = 22)		
Clinical trials (*n*=13)	Indication	EudraCT
RMS 2005	Rhabdo/non-rhabdomyosarcoma	2005-000217-35
LCH-IV	Langerhans cell histiocytosis	2011-001699-20
SIOP-PNET 5	Medulloblastoma	2011-004868-30
Inter-B-NHL-RITUX 2010	B mature non-Hodgkin lymphoma	2010-019224-31
OMS/DES 2011	Opsoclonus-myoclonus neuroblastoma	2011-000990-29
ESMART	All relapsed cancers	2016-000133-40
VINILO	Low grade glioma	2012-003005-10
BIOMEDE	Diffuse intrinsic pontine glioma	2014-001929-32
MEMMAT	Medulloblastoma	2010-023691-33
BEACON	Neuroblastoma	2012-000072-42
CELYVIR	All solid tumors	2019-001154-26
CRISP	ALK aberrant tumors	2015-005437-53
VERITAS	Refractory high-risk neuroblastoma	2015-003130-27

Observational studies/registries (*n* = 9)	Indication	EudraCT
SEHOP-LAL PETHEMA 2013	Acute lymphoblastic leukemia	–
AML 2007	Acute myeloid leukemia	–
ALL 2005	Acute lymphoblastic leukemia	–
ALL relapsed 2008	Relapsed acute lymphoblastic leukemia	–
LNH-SEHOP 2018 registry	Non-Hodgkin lymphoma	–
LNH-B04 database	B mature non-Hodgkin lymphoma	–
ALCL 99 database	Anaplastic large cell lymphoma	–
EURO-LB-02 database	Lymphoblastic lymphoma	–
ALL-2002	Acute lymphoblastic leukemia	–

### Time and recruitment metrics

Sixteen international clinical trials received integral support from ECLIM-SEHOP. In these studies, SEHOP is acting as a national co-sponsor and the principal investigator is serving as the national coordinator. Out of these, eight have active recruitment, one has been closed (*EURONET PHL-C2*), and seven trials are currently under set-up process. In these studies, a total of 584 patients have been recruited, out of which 54 patients are newly enrolled in the last 6 months from the observation period for this analysis.

**Fig. 3:**
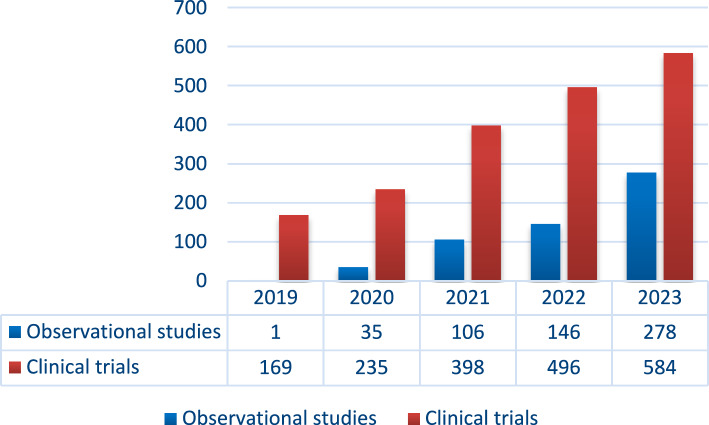
Cumulative patient recruitment in ECLIM-SEHOP clinical trials and observational studies

ECLIM-SEHOP supervises integrally nine observational studies/registries with comprehensive management. These studies have collected data on a total of 278 patients, including 55 newly enrolled patients in the last 6 months (data excluding imaging platform). Cumulative patient recruitment is shown in Fig. 3. Among these studies, seven are currently in recruitment phase, while two are undergoing administrative processes. Until now, 392 images have been undertaken on the SEHOP-CLOUD imaging platform, including 25 new cases in the last 6 months from the observation period for this analysis.

To measure the timings of the opening procedures, the available data from the nine integrally supported clinical trials containing recruitment was required from the CRO. Updated metrics for these timelines of clinical initiation are shown in Table 3.

**Table 3:** ECLIM-SEHOP integrally supported updated trial set-up metrics

Metric	Median (range), in months
Time from submission to AEMPS approval	3.0 (1.8–4.6)
Time from AEMPS submission to AEMPS approval	3.2 (0.6–6.0)
Time from CEIm approval to first site open in Spain	8.8 (3.5–41.8)
Time from AEMPS approval to first site open in Spain	7.1 (1.2–38.5)
Time from CEIm approval to > 50% of the sites open	12.6 (4.8–46.7)
Time from AEMPs approval to > 50% of the sites open	12.1 (2.5–43.4)
Time from first site open to first patient recruited	1.8 (0.1–12.6)
Time from CEIm submission to first patient recruited	13.3 (8.7–49.3)
Time from AEMPS submission to first patient recruited	12.2 (8.7–47.0)
Time from first international site open to first Spanish site open	31.3 (7.0–62.0)

*CEIm* Comité ético de investigación médica (medical investigation ethics committee), *AEMPS* Agencia Española de Medicamentos y Productos Sanitarios (Spanish competent authorities)

Including the 25 studies (clinical trials, observational studies and patient registries) that receive integral support and the 22 studies that have partial support, a total of 5240 patients have been recruited under ECLIM-SEHOP in international studies.

## Discussion

The creation and development of ECLIM-SEHOP platform has been a key milestone in promoting the treatment of childhood cancer by means of academic clinical trials in Spain. The studies included have increased exponentially and cover all pediatric malignancies and transversal topics.

The implementation of international trials has strengthened the Spanish pediatric oncology network, generated standard operational procedures and unified clinical, imaging and pathology criteria. Complying with the requirements of international sponsors, as many of the trials demand central imaging and pathology review, national hubs have been created for different cancers in various disciplines. As an example, central imaging review using SEHOP-CLOUD imaging infrastructure is unifying radiological interpretation in the *SIOP Ependymoma II* trial and this platform is also widely used in Hodgkin and non-Hodkin lymphoma protocols and other solid malignancies. Some national tumor groups such as the brain or the soft tissue sarcoma group also discuss complex cases in videoconference based tumor committees sharing radiological images by means of this facility and pediatric radiologists of different centers are being included in the decision-making procedures.

Regarding pathology, many of the studies such as *FAR-RMS, Euronet-C2, Umbrella, LOGGIC-CORE,* and others require centralized pathology review and all the tumor samples are referred to a unique center trying to achieve an homogeneous interpretation and higher quality diagnostic criteria [16]. Samples in some studies are further exported to an international hub for advanced genomic studies to secure that every patient has access to all the biologic determinations that the protocols demand.

One of the current challenges is the set-up of the academic phase III, *ALL-Together* platform for the treatment of acute lymphoblastic leukemia (ALL). This study is an initiative where several study groups from European countries including NOPHO, UKALL, DCOG, COALL, BSPHO, SHOP, and SFCE, previously collected their experience of successful treatment of children and young adults with ALL and designed a collaborative protocol. This new platform study is both a comprehensive system for stratification and treatment of ALL as well as the basis for several randomized trials included in the study design [17]. Implementing this study is a challenge per se as it targets pediatric and young adult front-line acute lymphoblastic leukemia expecting a high recruitment rate and including several sub-studies.

Other important observational studies and registries focused on key transversal aspects have been integrally supported by ECLIM-SEHOP. The *SACHA* study (*Secured access to innovative medicines for children, adolescents and young adults with cancer)* has been opened recently in four centers (H. La Fe, H. Miguel Servet, H. Dr. Balmis de Alicante and H. 12 Octubre) and will be active in 15 in the following months. The aim of *SACHA* is to secure the access of children with pediatric malignancies, non-eligible to clinical trials, to innovative therapies with compassionate use or off-label administration of anti-cancer drugs, following the SACHA-France study [18]. Focused on other issue, the *Survivorship Passport* study is looking toward the implementation of the follow-up of late effects of childhood cancer survivors (SIOPE survivors passport project) [19].

Significant progress has been observed regarding the set-up study metrics. Compared to the previous review in 2019 [13], the median time from competent authorities’ approval to the first site opening has been reduced by 2 months over the last 4 years. This reduction is indicative of an increasing inclusion of the required information in submissions and in cases where it is lacking, issues can be promptly corrected due to the smooth communication with the authorities. Additionally, the time from first site opening to the first patient recruited has decreased to 1.8 months. This reflects greater efficiency in the recruitment process, likely attributable to the strong commitment of Spanish sites. All these improvements have positioned Spain around the European average in terms of trial initiation timings [20, 21] revealing a significant opportunity to strengthen the country's position in pediatric cancer clinical research.

One of the most important metrics that summarizes the main objectives of ECLIM-SEHOP is the median time from the first international site open to the first Spanish site open. This duration has been significantly reduced from 41.5 to 31.3 months [13], reflecting the improved performance in delivering clinical research. However, the lengthy process of setting up international trials still exists and further work must be done to reduce the opening times of the trials in Spain after the opening in the leading country. Additionally, the adaptations to implement the new Clinical Trial Regulation [22], including the CTIS (Clinical Trials Information System) database managed by the European Medicines Agency (EMA), have produced delays in the opening of some of the trials. Despite this, it will soon allow sponsors to apply for authorization of a new clinical trial in up to 30 countries at the same time, simplifying processes and enhancing the effectiveness of all clinical trials, particularly benefiting those carried out in the Member States.

Historically, Spain has not been able to participate in multiple international clinical trials because of lack of resources and infrastructure to adapt to modern regulatory standards and provide good quality data. For some other studies, the time to set up the trials has been so long due to administrative barriers that the trials were completed or nearly completed internationally by the time they were open in the country [13]. These issues have been reflected in 5-year survival rate figures, where Spain has lagged behind other northern and central European countries. However, comparing the current trends, Spain shows significant progress in survival data. Based on the EUROCARE-5 project (data from 1978 to 2007) [23], the 5-year survival rate in the country for all tumors excluding CNS malignancies was 3% inferior than the average of all European countries (79% vs 82%). However, according to the updated EUROCARE-6 data (data from 2000 to 2014), this gap between Spain and Europe has decreased to 0.1% (85.2% vs 85.3%) (Table 4). The global increase in European countries was 3.3%, while Spain has doubled this increase (6.2%). Considering the 2023 RETI-SEHOP report, survival in the country has reached 82% so presently it is possible that Spain is or will be soon above the European 5-year survival average [4]. We believe that the gradual incorporation of Spain into international clinical trials with the support of ECLIM-SEHOP has largely contributed in this improvement.

**Table 4:** 5-year observed survival comparing EUROCARE-5 and EUROCARE-6 data [5, 23]

	EUROCARE-5 (data 1978–2007)	EUROCARE-6 (data 2000–2014)
Spain (all tumors excluding CNS)	79%	85.2%
Europe (all tumors excluding CNS)	82%	85.3%
Spain (acute lymphoblastic leukemia)	81%	86.7%
Europe (acute lymphoblastic leukemia)	85.5%	89.9%
Spain (all cancers)	–	81.1%
Europe (all cancers)	–	81.3%

The main challenge of the platform is to ensure long-term sustainability. To enhance the capacity for innovation in the field of clinical research and strive toward the goal of potentially including every child and adolescent in a clinical trial, it is imperative for public institutions to secure increased funding and bolster their capabilities. This includes the development of more academic trials and the implementation of collaborative international plans. [13]. ECLIM-SEHOP has been supported with funds from competitive and non-competitive grants from philanthropic organizations and, therefore, with increasing complexity of clinical trials, is in constant need of identifying financial resources to make it sustainable [13].

## Conclusion

ECLIM-SEHOP has proven to be an essential tool, signifying a paradigm shift in pediatric oncology research within our country. Through this organization, standard treatments in Spain are transitioning toward European state-of-the-art diagnostic and therapeutic strategies. The platform supports many front-line treatments for children with cancer, playing a pivotal role in enhancing survival rates in pediatric cancer and aligning Spain's situation with that of other European countries.

## Data Availability

Not applicable.
